# Elephantiasis of the Scrotum and Labia Pudendi

**Published:** 1830-04-01

**Authors:** 


### XXXII.

### ELEPHANTIASIS OF THE SCROTUM.

A very good account of this curious disease is given in his recent work,* by Dr. Titley, who appears, during his practice in the West Indian Islands, to have witnessed a good many cases of the kind. Known under different names in the East, in the West, in the African and the American Continents, Elephantiasis was for some time considered to be alien from Europe, and never to originate in the colder regions of the north. This, however, is undoubtedly an error, for affections of the lower extremities, resembling in all essential particulars the Barbadoes leg, are not very infrequent in this island, and in other countries of the continent of Europe. There is at this moment in St. George's Hospital, an instance of the malady in a man who was born in the neigbourhood of London, and who never was out of England in his life. M. Biett, too, in his Clinical Lectures, relates, if our memory serve us, several cases where natives of France were thus affected. Dr. Titley has seen it in an inhabitant of the Orkney Islands, and "understands that it is prevalent in some parts

---

* On the Diseases of the Genitals of the Male.

of Ireland." The following is an accurate account of the invasion and progress of the disease.

"The attack of this disease is very sudden, and comes on without any premonitory symptoms. A person apparently in perfect health will be seized with severe rigors, attended with acute pain in the head, back, and loins, nausea, and sometimes vomiting. There is also pain in the inguinal glands of one side. Sometimes the right, at other times the left side is affected, but never both together; and the gland which is the seat of the disease in the first attack is generally affected in each succeeding one. The rigors, after continuing an uncertain period, varying from half an hour to two or three hours, are succeeded by pyrexia, in which the skin is intensely hot, more so indeed than in any other fever I have seen. The hot stage usually continues from twenty-four to forty-eight hours, and sometimes longer; during which the patient is often delirious. If the leg be the part affected, as is most generally the case, the pain in the inguinal gland increases; and, if the patient be a white person, there is an erysipelatous blush over the part extending down the thigh; but, if the patient be a negro, there is a slight tumefaction and hardness along the course of the absorbent vessels.

"Sometimes the inflammation is of a more phlegmonous character, and then the swelling in the thigh is more circumscribed, and often terminates in an abscess, which will require to be discharged. The leg swells, and is much inflamed; and as the inflammation increases, the fever abates, and soon goes quite off, whilst the tumefied gland subsides and regains its natural state. The leg continues swelled and inflamed for several days longer, then gradually diminishes, and the patient seems to have again regained his perfect health.

"Sometimes the local symptoms are apparent before the fever sets in; at other times they occur so nearly at the same time that it becomes difficult to say which precedes. The fever makes frequent returns, but at very uncertain intervals; sometimes only two, three or four times a year; in others, once in a month, or three weeks or oftener. It will sometimes return at the end of two or three weeks, and the next time not until three, four, or six months; but whenever it reappears, the disease affects the same part on which it chanced to fall the first time. On each accession of fever there takes place an effusion of lymph into the cellular membrane: the part affected remains swelled for a longer period after each attack. After several returns, the quantity of lymph effused, being greater than can be absorbed, becomes coagulated and ultimately organised, by which the limb or part becomes permanently enlarged, and puts on very much the appearance of being anasarcous, but the swelling does not pit or retain the impression of the finger so much or so long as in a dropsical case. The skin, as the disease advances, becomes rough and rugged."

Patients live for many years with an enormous leg or scrotum, and enjoy good health except during the occasional attacks of fever. The lower extremities are not the only parts affected; the arm, the scalp, the ears, the back of the neck, in some rare cases even the lower part of the spine or os coccygis are liable to be attacked. The scrotum is a very frequent and the penis an occasional seat of the affection, although Dr. Hillary makes no mention of either being subject to the complaint. Sometimes it seizes on the stomach, intestines, or brain, producing the same symptoms as when these organs are affected with acute inflammation. Like gout and rheumatism, Dr. Titley remarks that the disease is liable to sudden metastases, and cites two cases in confirmation of his statement. The first was that of a middle-aged female, who had both legs permanently enlarged. The disease suddenly quitted these parts and "fixed upon the lungs," producing dyspnœa, cough, expectoration, which at length was purulent, emaciation, and hectic which ultimately proved fatal. The skin of the legs during this state of things became loose and flabby. The other case was that of a man, one of whose legs Dr.

Titley amputated below the knee. It was nearly as large as one's body, with deep clefts at the instep and ulceration of the foot between the toes, profuse watery discharge, and so much pain as to deprive him of rest both night and day; the other leg was slightly enlarged. The man went on well for nearly three weeks and the stump was almost cicatrized, when the disease "suddenly leaving the remaining leg fixed upon the stomach, producing symptoms precisely similar to those which take place when gout affects this organ;" as in the former instance the integuments of the leg hung loose and flabby. By the aid of powerful stimulants the disease left the stomach and returned to the leg, again distending the loose integuments. Two or three days afterwards he had a second attack which proved almost instantly fatal.

We regret that no post-mortem examination would appear to have been made in either case, and we regret it more particularly in the last. Gout "in the stomach" is, like virtue, a thing much talked of but little seen. Not long ago we saw an instance of this same metastasis of gout "to the stomach," as evinced by the ordinary symptoms of prostration, hiccup, &c. On *dissection* the gout was not found in the stomach at all, but in the lungs; that is, there were all the appearances of a recent and violent peripneumony, the stomach being healthy. We suspect that the translations of rheumatism and gout are not so entirely understood, as our brethren of the empiric school, those who see little of morbid anatomy but practise by tradition, and believe in no wisdom but that of their ancestors, are apt to imagine. Under these circumstances we are sorry that Dr. Titley rests only on symptoms, in proving his statements respecting the metastasis of elephantiasis to particular organs. That a metastasis took place we have no doubt; the *quò* is the question.

"When the scrotum is the part affected, I apprehend that, after a certain time, the lymphatic vessels become so much enlarged and relaxed, that they continue constantly to pour out their contents, so that the tumour increases independently of the febrile attacks. Where the penis is affected as well as the scrotum, these parts enlarge together in an equal ratio; but if the scrotum only be affected, then the penis, as the scrotum enlarges, becomes drawn in, so as ultimately to disappear and become completely imbedded in the tumour; the prepuce being distended elongates, and opens by a navel-like aperture on some part of the anterior surface, or even at the very end of the tumour. There is no limit to the magnitude which tumours of this kind may acquire. The testicles at first may be plainly felt in their natural situation in the centre of the swelling, but in a more advanced stage they cannot be discovered in consequence of the great thickness of the intervening integuments. For the most part they are healthy; though they may be simultaneously affected with any other disease to which they are subject, without reference to this. Hydrocele of one or both tunicæ vaginales is a very frequent occurrence, and the disease may be complicated with hernia."

There are many remarkable cases of this disease on record. Dionis, Cheselden, Walther, and Morgagni make mention of affections of the scrotum that can only have been of this description; but Chopart relates the most remarkable instance of any, in the person of a negro of the Coast of Guinea, who had lived for twenty-two years in Martinique, was fifty years of age, and whose scrotum weighed forty pounds, and reached to his ankles. M. Raymondon removed a tumour of this kind weighing twenty-nine pounds, and the patient died in the course of six hours. Nubert de Lounes also removed one of thirty pounds weight from the celebrated Charles de la Croix, formerly minister for foreign affairs in France, and with success. Baron Larrey describes the disease under the name of sarcocele, and says that all the patients were also more or less affected with elephantiasis. In one individual seen by the Baron, an agricultural labourer from Upper Egypt, the scrotum was computed to weigh fifty pounds; and in different parts of that country M. Larrey witnessed ten or twelve others nearly as large. This able old surgeon removed such a tumour of an oblong form, weighing three pounds, from a cook in a convent of Capuchins in Grand Cairo. In a case related by Dr. Hendy, where the enlarged scrotum was twenty-four inches in length and six feet in circumference, and where the left leg was also slightly enlarged, the patient died from mortification of the part. Dr. Hendy states that five other cases had come within his knowledge, where the scrotum became much enlarged, sloughed, and left the testicles entirely denuded.

Passing over a case by Mr. Corse, and one of elephantiasis of the penis by Mr. Wadd, we may mention that one was published in the sixth volume of the Medico-chirurgical Transactions, in which our author amputated the enormous scrotum with success. Subsequently to this, our author assisted Dr. Caines in performing the operation on three patients, in one of whom the enlargement of the scrotum was complicated with hernia on the right side. All the patients recovered. Four other operations for elephantiasis of the scrotum by Dr. Titley were attended with a similar fortunate result.

"But the most remarkable tumour of this description, which I have either seen or heard of, was attached to a man belonging to the estate of the Rev. Mr. Verchild; and from this the late Mr. Wilkes endeavoured to separate the unfortunate possessor on the 6th February, 1815. I was accidentally prevented from being present at this operation, but the following particulars were communicated to me by Mr. Wilkes. The length of the tumour was two feet five inches; its circumference five feet ten inches; and its weight one hundred and sixty-five pounds avoirdupois. The operation occupied *nearly eight hours*, and the man died apparently from exhaustion towards its conclusion; a copious venous hæmorrhage followed each stroke of the knife; the lymphatic vessels were very much enlarged and were apparent, traversing the tumour. My friend Mr. Jordan, of Weymouth-street, at that time stationed in St. Christopher, as surgeon to the forces, was present, as were also Messrs. Richards and Waterson of the 15th regiment, and Dr. Clifton, a practitioner of the island. The operation was likewise viewed by several gentlemen not of the profession, and the tumour was seen by the Rev. Mr. Verchild and Mr. Goldfrap.

"I once assisted at an operation of this kind which terminated unfavourably. The tumour in this case measured in length twenty inches, and in circumference forty-four. The patient was a young man, and, although anxious for the removal of the tumour, yet he was under a state of great alarm, as was evident both from his countenance and manner. Notwithstanding the operation was performed with great dexterity and celerity, not having occupied half an hour, and the hæmorrhage was very trifling, yet the poor fellow most unexpectedly died on the table.

"Whilst these swellings are yet of moderate size the operation is comparatively easy; but when they have attained a magnitude approaching to that of my first case, then it becomes, probably, the most laborious piece of dissection that occurs in the practice of surgery."

Our author relates the particulars of two more successful operations, one by Mr. Liston, from the nineteenth volume of the Edinburgh Medical and Surgical Journal; the other by Dr. Wells of Maracaybo, Colombia, from the American Journal of Medical Sciences for May, 1828.

### ELEPHANTIASIS OF THE LABIA PUDENDI.

Baron Larrey observes, that no author with whom he is acquainted has spoken of a similar disease as affecting the female parts of generation, and accounts for it by supposing that the periodical evacuations of that sex prove their safeguard. The worthy Baron, however, adds that, as if by a frolic of Nature, a female of Grand Cairo, named Ammeh Fatoumy, furnished a well-characterized example of sarcocele of the labia pudendi. The Baron proposed extirpating the diseased parts, but, receiving a sudden order to join the army on its march to Alexandria, he was necessarily prevented from executing his design.

Dr. Titley has seen only one example of elephantiasis in the labia pudendi, and that was in a young negress. The parts were very much enlarged, and were amputated by Dr. H. M. Clifton of St. Kitt's; they weighed one pound —no hæmorrhage took place, not a vessel was secured, and the wound healed kindly.

A third case was operated on by Mr. Liston. The woman, named Smith, was about thirty years of age, and applied to Mr. L. on account of a large tumour attached to the labium. It had been growing four or five years, and from its size had at length rendered her almost incapable of following her employment. The removal was attended with considerable difficulty, on account of its neck extending along the vagina for some distance into the pelvis. The attachments of the tumour to the sphincter vaginæ were so strong as to be separated with difficulty, and, after this was accomplished, the contraction of the orifice was very remarkable. The tumour weighed upwards of ten pounds. The case terminated very favourably, and the woman now enjoys perfect health.

This brings our notice of the chapter on elephantiasis of the scrotum and labia pudendi to a close. Our author has evidently seen much of a disease which is perfectly new to many medical men in this country, and which is more often met with, no doubt, than understood. If he makes no mention of any curative means, except the knife, it is probably because he knows of none, and such a proceeding is more honest than frequent. In the elephantiasis of the extremities compression is certainly useful in some cases, but such a plan of treatment is inapplicable, or nearly so, to the scrotum. The knife would appear to be the only effectual remedy, and the number of successful cases that we have mentioned, hold out a very favourable prospect to the operator and the patient. It is only in the *very* great enlargements of the scrotum, that serious or fatal consequences would seem to have followed the operation.

